# Transparent Electrodes Based on Silver Nanowire Networks: From Physical Considerations towards Device Integration

**DOI:** 10.3390/ma10060570

**Published:** 2017-05-24

**Authors:** Daniel Bellet, Mélanie Lagrange, Thomas Sannicolo, Sara Aghazadehchors, Viet Huong Nguyen, Daniel P. Langley, David Muñoz-Rojas, Carmen Jiménez, Yves Bréchet, Ngoc Duy Nguyen

**Affiliations:** 1 Univ. Grenoble Alpes, CNRS, Grenoble INP (Institute of Engineering Uni. Grenoble Alpes), LMGP, F-38000 Grenoble, France; melanie.ml.lagrange@gmail.com (M.L.); Thomas.SANNICOLO@cea.fr (T.S.); sara.aghazade@gmail.com (S.A.); viet-huong.nguyen@lmgp.grenoble-inp.fr (V.H.N.); david.munoz-rojas@grenoble-inp.fr (D.M.-R.); carmen.jimenez@grenoble-inp.fr (C.J.); 2 Univ. Grenoble Alpes, CEA, LITEN, F-38054 Grenoble, France; 3 Département de Physique, Université de Liège, CESAM/Q-MAT, SPIN, B-4000 Liège, Belgium; ngocduy.nguyen@ulg.ac.be; 4 CEA-INES, LITEN, 50 Avenue du Lac Léman, F-73375 Le Bourget-du-Lac, France; 5 ARC Centre of Excellence for Advanced Molecular Imaging, Department of Chemistry and Physics, La Trobe Institute for Molecular Science, La Trobe University, Melbourne, Victoria 3086, Australia; d.langley@latrobe.edu.au; 6 Univ. Grenoble Alpes, CNRS, Grenoble INP, SIMAP, F-38000 Grenoble, France; ves.BRECHET@cea.fr

**Keywords:** metallic nanowire, transparent electrode, network, silver nanowire, percolation, stability, flexible, (Spatial) ALD.

## Abstract

The past few years have seen a considerable amount of research devoted to nanostructured transparent conducting materials (TCM), which play a pivotal role in many modern devices such as solar cells, flexible light-emitting devices, touch screens, electromagnetic devices, and flexible transparent thin film heaters. Currently, the most commonly used TCM for such applications (ITO: Indium Tin oxide) suffers from two major drawbacks: brittleness and indium scarcity. Among emerging transparent electrodes, silver nanowire (AgNW) networks appear to be a promising substitute to ITO since such electrically percolating networks exhibit excellent properties with sheet resistance lower than 10 Ω/sq and optical transparency of 90%, fulfilling the requirements of most applications. In addition, AgNW networks also exhibit very good mechanical flexibility. The fabrication of these electrodes involves low-temperature processing steps and scalable methods, thus making them appropriate for future use as low-cost transparent electrodes in flexible electronic devices. This contribution aims to briefly present the main properties of AgNW based transparent electrodes as well as some considerations relating to their efficient integration in devices. The influence of network density, nanowire sizes, and post treatments on the properties of AgNW networks will also be evaluated. In addition to a general overview of AgNW networks, we focus on two important aspects: (i) network instabilities as well as an efficient Atomic Layer Deposition (ALD) coating which clearly enhances AgNW network stability and (ii) modelling to better understand the physical properties of these networks.

## 1. Introduction

A considerable amount of research has been devoted lately to nanostructured transparent conducting materials, which play a pivotal role in many modern devices [1,2,3,4] such as solar cells [5,6], flexible light-emitting devices [7], touch screens, electromagnetic devices, and flexible transparent thin film heaters [8,9,10,11]. Currently, the most commonly used material for such applications, ITO (indium tin oxide), suffers from two major drawbacks: indium scarcity and brittleness. As a consequence, several emerging transparent electrodes (TE) have been studied lately including grapheme [3], carbon nanotubes [3], metallic grids [12], and metallic nanowire (MNW) networks [13,14,15]. Silver nanowire (AgNW) networks appear to be promising substitutes for ITO since these percolating networks exhibit excellent properties with sheet resistances of a few Ω/sq associated to optical transmittances of 90% [6,16], fulfilling the requirements for many applications [14]. Bending tests clearly show that MNW networks also exhibit very good electro-mechanical properties [13] which are key assets for addressing emerging flexible electronics applications. In addition, the fabrication of these electrodes involves low-temperature process steps and upscaling methods, such as spray deposition, thus making them very appropriate for future use as TE in flexible device fabrication compatible with, for instance, roll-to-roll processing [17]. 

In spite of all of these positive characteristics, thorough investigations devoted to MNW networks should still be performed to make them really competitive with more standard Transparent Conductive Oxides (TCO), which have been studied for several decades [1,2,18,19,20] and are still widely used in industry. For instance, the prevalent potential weakness of MNW networks so far appears to be their possible lack of stability, which is associated with thermal instability [16,21], electrical failure [22], or ageing [23]. Besides Ag nanowires, it could be strategically interesting to consider cheaper metals such as Cu (or CuNi) nanowires [24,25,26,27]. However, growth and stability appear more problematic for such MNWs at present. 

The influence of network density, nanowire size, and post-deposition treatments on the physical properties of MNW networks are discussed in the present contribution. Network density (as well as the useful notion of areal mass density) is a crucial parameter [28,29] which predominantly determines both the electrical conductivity and the optical transmission of the network. A sparse network is very transparent, but correspondingly has a high electrical resistance, while a dense network is very conductive, but appears opaque or hazy; therefore a trade-off has to be considered [16]. The optimal combination of density and transparency (including haziness) needs to be determined by the requirements of the final application. The stability of MNW networks, both electrical and thermal, is also briefly discussed. This is indeed a crucial aspect for the efficient integration of MNW networks in devices. Lately, nanocomposites fabricated with MNWs and an embedding matrix or coating (either polymer or oxide) appear promising since they can potentially offer new properties and enhanced stability. Nanocomposites appear to be more stable than bare MNW networks [30,31]. Finally, let us also mention the use of MNW networks for medical applications, such as thermotherapy [30] and the integration of MNW networks in textiles [11,32]. 

Numerical approaches are useful for better understanding or even predicting the properties of MNW networks [9,33,34]. Monte Carlo simulations have been used to investigate the percolation onset within MNW networks (more generally called stick percolation [35,36,37]), while multiscale simulation appears very useful for better understanding the properties of MNW networks [38,39]. 

This work aims to present the main properties of MNW networks (part 2), then network stability is considered and a method to enhance it is presented in part 3, while simulations and semi-empirical modelling of MNW networks are discussed in part 4, to better understand their physical properties. 

## 2. Properties of Metallic Nanowire Networks

The prevailing physical properties of a TCM are optical transparency and electrical resistance, although other properties are to be considered as well (such as haziness, roughness, stability, mechanical properties, energy band alignments, etc.).

**Figure 1:**
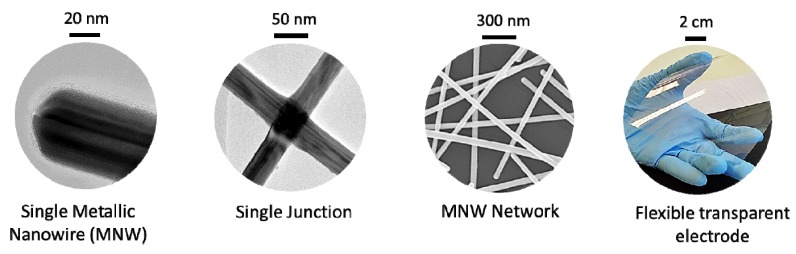
The main length scales at play in metallic nanowire (MNW) networks. From left to right: Transmission Electron Microscopy observations of the extremity of a single MNW and of a junction between two adjacent MNWs; Scanning Electron Microscopy observation of a dense MNW network; picture of a macroscale flexible transparent electrode composed of MNWs.

The macroscopic physical properties of a specimen composed of a MNW network depends on the network density and the physical properties of the nanowires themselves. Figure 1 shows the main length scales which need to be considered in order to understand the properties of MNW networks. 

Several methods can be used to deposit MNW networks. Table 1 below briefly compares the characteristics related to homogeneity, scalability, and cost associated to some of these methods that can be found in the literature.

The five techniques reported in Table 1 are all compatible with low temperature deposition processes and do not require vacuum processing chambers (pressure < 1 mbar), both of which are clear advantages compared to deposition methods associated with state of the art TCO specimens. Table 1 describes general trends since many parameters have to be considered such as application requirements (searched prevailing properties, cost, etc.), MNW morphology, ink formulation, or experimental conditions. However, some general comments can be stated. Spin coating appears to be an easy and fast method, but is not scalable and exhibits non-homogenous orientation distribution of AgNWs. Spray coating appears to have several advantages: very easy surface scalability, reproducibility, and can be used with masks. In principle, spray coating can be used on different substrates and is appropriate for large volume deposition. Vacuum filtration appears to be very homogeneous and does not require large quantities of solution, but appears to be poorly surface scalable. Drop casting is not scalable and may show spatial non-homogeneities (such as coffee rings) compared to other methods, but can be an easy method for quick tests. Finally the Meyer rod method is very simple and compatible with roll-to-roll fabrication [15], however appropriate ink formulation should be considered with constraints related to ink viscosity. 

The macroscopic properties of MNW networks drastically depend on the local properties at the nanowire scale. For instance, the electrical resistivity of an individual MNW, $\rho_{NW}^{Ag}$, is a function of the wire diameter and of the resistivity of bulk silver, $\rho_{bulk}^{Ag}$, as shown by Bid et al.[41]:

(1) $$\rho_{NW}^{Ag}=\rho_{bulk}^{Ag}\cdot (1+\frac{\Lambda }{2\cdot D_{NW}})$$

where Λ is the mean free path of the electrons in bulk silver and *D_NW_* is the average diameter of the MNWs. When *D_NW_* approaches Λ (in pure bulk silver, Λ is roughly equal to 40 nm) then electron scattering occurring at the lateral surfaces of the MNWs plays a dominant role in the conduction mechanisms and leads to an increase in the electrical resistivity of the wire. Therefore, the thinner the MNWs, the larger their electrical resistivity. As a consequence, when a current flows through a MNW, the amplitude of local heating due to the Joule effect will be larger since the local heating power per unit volume can be written as $\rho_{NW}^{Ag}$·*j*^2^ where *j* is the current density. This should be considered in parallel with the dependency of ampacity (i.e., the maximum current density that a MNW can be subjected to before failure) [42] with MNW diameter, as well as the lower thermal stability observed in smaller MNWs, as seen below. 

Lagrange et al. demonstrated that when subjected to a thermal ramp, the temperature at which a MNW network loses stability, causing the onset of an increase of the resistance, depends upon *D_NW_* in the following form: [16]

(2) $$T_{s}(D_{NW})=T_{s}(\infty )\cdot (1-\frac{C}{D_{NW}})$$

where *T_s_*(∞) denotes the characteristic temperature of bulk [16], and *C* depends on the considered metal through the values of its bulk molar volume ($V_{m}^{S}$), free surface energy ($\gamma_{S}$), and the molar latent heat of melting (*L*) through the following equation: $C=V_{m}^{S}\cdot \gamma_{S}/L$.

**Figure 2:**
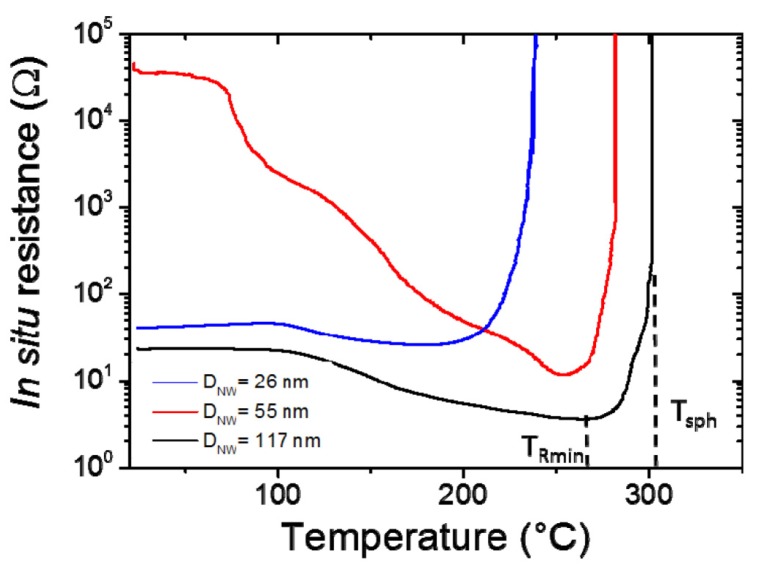
Evolution of the electrical resistance (*R*) of networks deposited by spin-coating and composed of AgNW with different average diameters (*D_NW_*) during a 2 °C·min^−1^ thermal ramp in air. One can clearly observe that the smaller the diameter, the lower the characteristic temperature, in agreement with Equation (2).

In order to significantly improve metallic nanowire-based transparent electrodes, thermal annealing of the as-deposited MNW networks is generally used since it has been shown to induce a significant decrease of the contact resistance between nanowires [7,16,21,43,44,45]. This is illustrated in Figure 2, where the in-situ electrical resistance of AgNW networks is plotted versus temperature during an annealing with a ramp of 2 °C/min for three different types of AgNW associated to average diameters of 26, 55, and 117 nm. Figure 2 shows the significant impact of thermal annealing on electrical resistance, which can decrease by several orders of magnitude [16,21,45]. The different regions of the curves can be interpreted as follows. First, in the low temperature area (below 200 °C), the decrease in resistance is associated with the thermal desorption of organic residues which are present around the AgNW and at their junctions. More precisely, these organic compounds are residuals left from the solvents used to disperse the nanowires for deposition, as the deposition is often performed by spin coating at room temperature. Isopropanol acts as an insulating layer when it is trapped between two nanowires by capillarity, therefore its removal leads to a more intimate connection and better electrical contact between nanowires. A second stage of resistance reduction at higher temperature is visible in Figure 2, after which a minimum is reached. This is ascribed to the optimization (on average) of the local sintering at the nanowire junctions thanks to a thermally induced surface atomic diffusion [21]. This phenomenon is driven by the reduction of surface energy of the nanowires. The minimum resistance is achieved when the welded junctions lead to the creation of enough efficient percolating pathways across the network. Eventually, at higher temperature, the network resistance grows without bounds within an increase of a few degrees from a typical point called the spheroidization temperature. This is associated to the nanowires’ morphological instability, driven again by surface energy reduction. Such a phenomenon corresponds to the Plateau-Rayleigh instability, a well-known phenomenon observed for nanowires [46]. It is also worth noticing that alternative post-deposition methods to thermal annealing can be used efficiently to optimize MNW network properties: laser sintering [47], light induced plasmonic nanowelding [48], and mechanical pressing [43].

Figure 2 also illustrates the effects of the AgNW diameter on the behaviour of the AgNW networks. Both the spheroidization temperature (*T_sph_*) and the temperature at which the minimum resistance is observed for each sample (*T_Rmin_*) increases with the nanowire diameter. *T_sph_* was considered to occur when the network resistance was above 10^6^ Ω, after the minimum resistance was achieved. The data shown in Figure 2 is thus fully consistent with Equation (2) [16].

One way to optimize MNW networks is to perform a thermal ramp, such as those reported in Figure 2, on a sacrificial specimen and then to perform the same thermal ramp on a fresh equivalent specimen up to the temperature where the electrical resistance was observed to be minimal. Such a thermal ramp up to *T_Rmin_* can be supposed to be a rather optimized thermal treatment.

It is worth noticing that the two combined physical features described above by Equations (1) and (2) indeed show that the smaller the average diameter of the nanowires, the lower their electrical stability.

**Figure 3:**
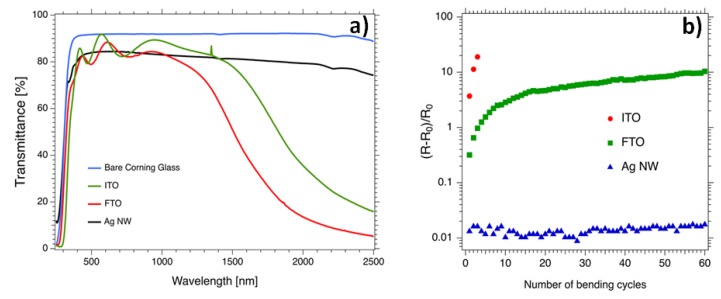
(**a**) Optical total transmittance (substrate contribution non-subtracted) of a bare substrate (in blue) and three types of transparent conductive materials in the UV-VIS-NIR region: Indium-doped Tin Oxide (ITO, green), Fluor-doped Tin Oxide (FTO, red), and AgNW network deposited by spray coating (black) are depicted. The associated sheet resistance is reported and is 15, 16, and 11 ohms per square for ITO [49], AgNW network [16], and FTO [50], respectively. (**b**) Comparison showing the relative increase of the electrical resistance between opposite sides of different transparent conductive materials TCMs when subjected to mechanical bending. The associated bending radius of curvature is equal to 5 mm while the TCM is on the opposite side to the bending radius and therefore placed under tension (data extracted from Sannicolo et al. [14]).

In order to place AgNW networks in the broader context of TCM to gain perspective, a comparison with more standard TCM based on oxides is necessary. Figure 3a shows the total transmission in the UV-VIS-NIR spectra of several types of TCMs, namely, Fluorine-doped Tin Oxide (FTO) and Indium-doped Tin Oxide (ITO), two well-known TCOs [1,2] that have been investigated for several decades, and AgNW networks for comparison. These three types of TCMs exhibit rather similar averaged optical transmission in the domain (400–1000 nm) and have comparable sheet resistance values: their associated sheet resistances are 15, 16, and 11 Ω/sq for ITO [49], AgNW network [16], and FTO [50], respectively. However, several differences can be noticed: (i) thickness fringes are clearly observed in the visible range associated to the continuous nature of the thin TCO layer, (ii) the AgNW network exhibits a much higher transmittance in the near Infra-Red (NIR) range since the gaps between AgNWs are fully transparent.

As shown by Figure 3a, AgNW networks are already capable of matching more conventional TCO in terms of optical and electrical properties [14,28]. However, the final choice of the optimal TCM for a given application will also depend on many other parameters, for instance: temperature fabrication, cost, stability, scalability, and bendability. The latter characteristic has been compared in Figure 3b for the same types of TCMs during repeated bending tests with a radius of curvature of 5 mm. ITO and FTO have a thickness equal to, respectively, 300 and 250 nm (see more details in part 5: Materials and Methods). ITO appears to be less compatible with the bending tests since the associated electrical resistance is multiplied by a factor of 12 after only two bending cycles. The associated resistance of the FTO layer is slowly increased with the number of bending cycles while the one associated to the AgNW network is only increased by a few percent even after 60 repeated bending cycles, showing good response under the bending tests. Such flexible properties have been used in flexible light-emitting electrochemical cells in which an AgNW network was embedded in a polyurethane optical adhesive to serve as a transparent electrode [51].

It is also worth noting that other types of mechanical tests have been performed to assess the mechanical response of MNW based transparent electrodes. For instance, stretching tests reveal that the AgNW networks can retain very good optical and electrical properties. This has been used to maintain the electroluminescent performances of AgNW-based elastomeric polymer light-emitting diodes, which are unaffected when exposed to deforming strains [52] and thus pave the way for stretchable Organic Light-Emitting Diodes (OLED) displays.

## 3. Towards the Integration of MNW Networks into Real Devices: Study of Stability of MNW Networks

While the optical properties (transparency and haze factor) and electrical resistance are the prevailing characteristics for TEs, several other characteristics, such as adherence, mechanical properties, spatial homogeneity, and stability, play a key role in some applications [14]. We now focus the discussion on the stability of MNW networks, which can be a serious issue when the integration of MNW networks in industrial devices is considered.

Several kinds of stabilization issues, such as electrical [22] and thermal stability [21], chemical stability, or ageing in standard conditions of operation [23,53], have been the subject of several studies. It has already been mentioned above that at high temperatures, due to surface energy reduction, MNWs tend to spheroidize [21]. This morphological instability is accompanied by the loss of the network connectivity, leading to a resistance increase, as shown in Figure 2. The dependence of the temperature associated to the spheroidization with the AgNW diameter should then be considered for the integration of MNWs into any device. 

Generally speaking, the origin of any MNW instability (electrical or thermal) that results in early failure, is linked either to silver atomic surface diffusion, electromigration processes, or a modification of the surface chemistry. One direct way to prevent such instability is to embed the MNW in a thin shell forming a nanocomposite. For instance, the use of reduced graphene oxide to coat either AgNW [54] or CuNW [55] has resulted in a much better stability. Transparent oxides can also be efficiently used for such a coating. For instance, Morgenstern et al. used ZnO nanoparticles as a shell for a AgNW network used as TE for organic solar cells [5], while Göbelt et al. encapsulated AgNWs with an Al-doped ZnO layer thanks to the atomic layer deposition (ALD) method [40]. 

**Figure 4:**
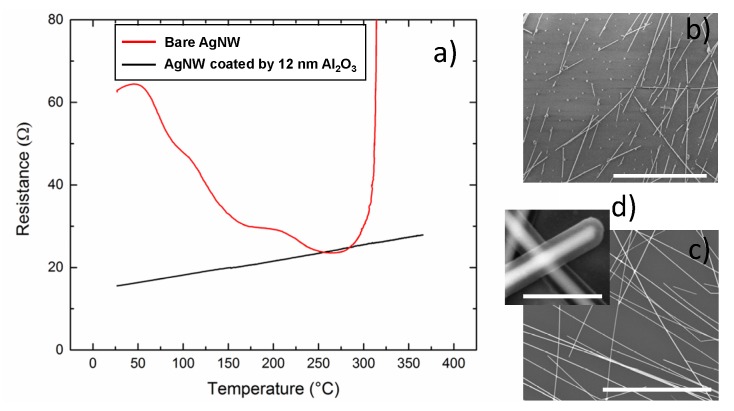
(**a**) In-situ measured resistance versus temperature of a AgNW network (deposited by spray-coating) during a thermal ramp showing that when a 12 nm thick layer of Al_2_O_3_ is deposited by SALD on AgNWs, a clear stability enhancement is observed since the network can undergo a thermal ramp up to 315 °C for bare AgNW and at least up to 370 °C for coated AgNW. For the coated network, the metallic behaviour can be observed through the linear increase of the electrical resistance with temperature associated to electron-phonon interactions. The SEM pictures are associated to bare AgNW (**b**) and coated AgNW (**c**) with 12 nm of Al_2_O_3_. The SEM picture (**d**) corresponds to a higher resolution of a AgNW network coated with 43 nm of Al_2_O_3_, where the coating can be clearly seen. Scale bars are 10 µm for (b) and (c) and 500 nm for (d). AgNWs have an average diameter of 90 nm and lengths between 10 to 20 µm, and the associated *amd* value of the network is about 85 mg/m^2^.

The unique properties of ALD make it a very suitable method to nano-engineer the surface of nanowires. A recently developed approach to ALD, Spatial Atomic Layer Deposition (SALD) [56], combines the advantages of ALD with several assets such as rapid and conformal growth of thin layers, and it is possible to implement it in atmospheric conditions and even in the open air [56]. SALD is thus particularly suited to be combined with TE based on AgNWs since it offers scalability and high throughput. An example of its use to enhance MNW network stability is reported in Figure 4a, where AgNWs were coated with a thin layer of Al_2_O_3_. The network was first annealed for 1 h at 230 °C and then a 12 nm thick coating of Al_2_O_3_ was deposited by SALD at a temperature of 200 °C using a home-made system described elsewhere [57] and trimethyl aluminium and water as precursors. The electrical resistances of the coated sample and of a similar uncoated AgNW network were measured during a thermal ramp of 2 °C/min up to 370 °C. The red curve is associated with the bare AgNW network, and is equivalent to the ones presented in Figure 2; the observed resistance decrease is associated first with desorption and then with local sintering at the junction, while the divergence at larger temperature corresponds to the morphological instability of AgNWs discussed above. For the coated AgNWs, the initial decrease in resistance is not observed since the thermal annealing at 230 °C and the SALD deposition at 200 °C leads to an already efficient local sintering. The preparation of the ZnO coated nanowire network via ALD enables the formation of a well sintered (optimal inter wire connections) AgNW network prior to its encapsulation with Al_2_O_3_. The electrical resistance of such a treated network is thus expected to be similar to a metallic sample, where the linear increase of resistance with temperature is due to the increased scattering from the electron-phonon interaction [42]. As shown in Figure 4a, a clear thermal stability enhancement was observed with the Al_2_O_3_ layer, since the coated AgNW network does not show any evidence of instability during the whole thermal ramp (see the black curve). SEM observations after the thermal ramp are reported in Figure 4b,c for the bare and coated AgNW, respectively. The bare NW network clearly exhibits deteriorated AgNW, while the coated ones appear unchanged. A SEM image at higher magnification is reported in Figure 4d; it concerns a similar network for which 24 nm of Al_2_O_3_ were deposited. One can clearly observe the typical highly conformal Al_2_O_3_ coating around the AgNWs expected for an ALD or SALD coating. While the oxide coating effectively protects the NW, a careful choice of the oxide needs to be considered in order to limit losses in transparency. For instance, the very thin Al_2_O_3_ coating used in the experiment reported in Figure 4 induces a very low decrease of the optical transparency, since the optical transparency of bare and Al_2_O_3_ coated MNW networks are 92.4% and 91.4%, respectively, at a wavelength of 550 nm. 

In conclusion, nanocomposites made of MNW and a thin oxide layer clearly exhibit higher stability, although such an approach leads to much broader consequences than only stability improvement: it can also come with improved interface issues such as adhesion, roughness, balance of work functions, etc. Therefore, fabricating such nanocomposites can also provide a wider range of opportunities in terms of device integration. For instance, MNW deposited on textiles can be used safely for human portable applications [11,32]. MNW based-flexible heaters have also proven to be compatible with medical thermotherapy [30]. Fabricating nanocomposites based on MNWs and other materials (polymers, carbon based materials, or oxide) paves the way to innovative solutions for many devices. This is definitely a critical direction for the future of MNW integration. 

## 4. Modelling of MNW Networks: A Brief Survey

One of the most important parameters in percolation theory is the network density, *n*, defined as the number of nanowires per unit area. The critical density, *n_C_*, is defined as the network density of an infinite network where the probability of finding a conduction path from one side to the other (i.e., the probability of percolation) is 1/2. Monte Carlo simulations are an efficient numerical tool to link *n_C_* and the average MNW length, *L*. It has been found that *n_C_* and *L* are related through the following expression [36]:

(3) $$n_{C}≅5.64/L^{2}$$

**Figure 5:**
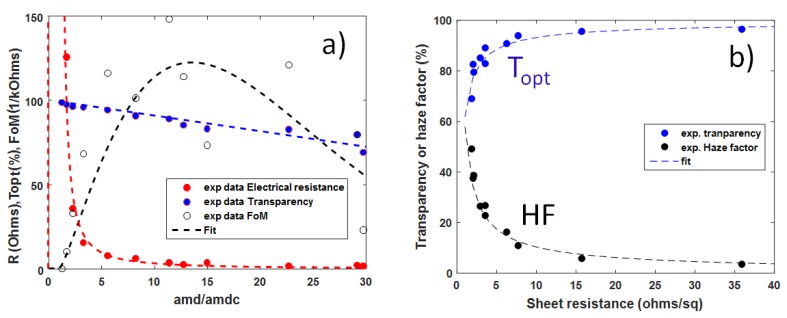
Properties of networks with AgNW of average diameter 117 nm and average length 42.5 µm deposited by spin-coating. (**a**) Dependence of physical properties versus reduced areal mass density (*amd*/*amd_c_*) of AgNW networks: minimum electrical resistance measured during thermal ramp (as that shown in Figure 2) (*R_min_*), optical transparency (*Topt*), and haze factor (*HF*), the later two measured at a wavelength of 550 nm. The circles are experimental data while the fits correspond to the use of Equations (5) and (6) for the resistance and transparency, respectively; (**b**) Optical transmittance and haze factor (*HF*) versus sheet resistance of the networks made of AgNW117 networks with various network densities. The fits correspond to the use of Equations (7) and (10), for the transparency and haze factor dependence on sheet resistance, respectively.

For practical reasons, it can be very convenient to consider the areal mass density, *amd*, instead of the network density (*amd* is simply *n* times the average mass of a MNW) and is often expressed in mg/m^2^. Below the critical density, *n_C_*, there is, on average, no percolating pathways and therefore the associated electrical resistance can be considered as infinite. Sparse networks have not been intensively studied so far, and while they are interesting from the fundamental viewpoint [45], their associated electrical resistance is too large for most applications. For networks with a density of a few *n_C_* (i.e., not too dense networks) the percolative regime comes into play and the electrical conductivity σ is then given by the following dependence on *n* or *amd*:

(4) $$\sigma \propto {\left(n-n_{C}\right)}^{\gamma }\;or\;\sigma \propto {\left(amd-amd_{C}\right)}^{\gamma }$$

where γ is equal to 4/3 for a two dimensional network [28,29,33]. Therefore if one considers that the contact resistance is negligible compared to the resistance associated to the MNW network (which is clearly valid for sparse networks), then the sheet resistance dependence upon *amd* can be written as:

(5) $$R_{sh}=K\cdot {\left(amd/amd_{C}-1\right)}^{-4/3}$$

where *K* depends upon the geometrical specimen features but is not a function of *amd*. This dependence of the electrical resistance of a MNW network on *amd* is valid as shown by Figure 5a where the fit using Equation (5) is in good agreement with the experimental data obtained from the study of a series of AgNW networks of different densities. 

In parallel, the dependence of the optical transmittance versus *amd* appears to be a linear decrease [16,58,59]. This can be explained by shadowing effects [16]. Therefore the optical transparency versus *amd* can be written in the following way:

(6) $$T_{opt}^{0}=T_{opt}^{0}\cdot (1-\alpha \cdot amd/amd_{C})$$

where α is a coefficient that can be deduced from the experimental data as shown in Figure 5a and which is, for the studied AgNW type, equal to 9.3 × 10^−3^. $T_{opt}^{0}$ refers to the optical transmittance of the bare substrate. Both Equations (5) and (6) can be combined to eliminate *amd* and this allows one to directly link *R_sh_* and $T_{opt}^{rel}$ through the following equation:

(7) $$T_{opt}=1-\alpha \cdot (1+\frac{K}{{R_{sh}}^{3/4}})$$

where K is a constant. A fit of the experimental data with Equation (7) is reported in Figure 5b, where the total optical transmittance was measured at a wavelength of 550 nm. Although the considered model is simple, a good agreement is observed. 

It is also worth noting that the performances of an optimized transparent electrode composed of AgNWs are very good when compared to the other types of TEs [1,15,16]. Performances as good as *T_opt_* > 90% and *R_sh_* ≤ 10 Ω/sq have been reported several times. Such performances are compatible even with the most demanding applications in terms of optical and electrical properties, i.e., photovoltaic applications [14,60]. 

One way to determine the best trade-off between optical transparency and the electrical resistance is to maximize a figure of merit, FoM. The most widely used FoM, especially for solar cell applications, is the one proposed by Haacke [15,61]:

(8) $$FoM=\frac{{\left(T_{opt}\right)}^{10}}{R_{sh}}$$

By using the dependences of *R_sh_* and $T_{opt}$ with *amd* (Equations (5) and (6), respectively) one can readily determine the *amd* value that will maximize the *FoM* given by Equation (8):

(9) $$amd^{\max}=\frac{1}{17}(\frac{2}{\alpha }+15)\cdot amd_{C}$$

Therefore for the given AgNW morphology (here *L_NW_* = 42.9 µm, *D_NW_* = 117 nm), the network areal mass density that will maximise the electro-optical properties is about 13 times *amd_C_*, the latter corresponding to the onset of percolation. This could be experimentally assessed by studying the transmittance, sheet resistance, and FoM values of the networks made with AgNW networks of various densities. Figure 5a displays the experimental values of FoM. The rather scattered values originate, on the one hand, from the large incertitude related to the calculation of *amd* values (especially for the dense networks), and, on the other hand, from the fact that slight fluctuations from the optical transparency values lead to very large variations of the FoM values (due the power tenth of Equation (8)). As shown in Figure 5a, the sample leading to the best FoM has a ratio *amd/amd_c_* of 13.7, in good agreement with the calculation. 

Although silver is considered as an expensive material, such optimized AgNW based TE can be considered as cheap since it requires a rather low amount of silver. For instance, an *amd* value equal to 13 times *amd_c_* corresponds to a silver amount equivalent to a 20 nm thick homogeneous layer for the nanowires used above.

Choosing a small average AgNW diameter induces shadowing to a lower extent, and therefore leads to larger optical transmission values. However, we also know from Equation (1) that a AgNW diameter smaller than the mean free path of electrons (i.e., Λ in bulk Ag is close to 40 nm) will increase the electrical resistivity and will also increase the electrical instability issues. Therefore, the optimized AgNW diameter would be typically in the range from 60–100 nm and the optimized *amd* would be in the range of 5–10 times the *amd_c_* value, a smaller value compared to the one observed experimentally above, which was associated to a diameter of 117 nm. 

As well, the haze factor *HF*, defined as the proportion of the diffuse component of the total transmitted light, plays a key role for many applications. Several studies have been indeed devoted to the evaluation of HF in MNW networks. The HF has been shown to increase linearly with *amd* [16]. By writing *HF = δ*·*amd*/*amd_c_*, where *δ* is constant, the relation between *R_sh_* and *HF* can be deduced from Equations (6) and (7), as follows:

(10) $$HF=\delta \cdot (1+\frac{K}{{R_{sh}}^{3/4}})$$

Equation (10) has been used to fit the experimental dependence between *HF* and *R_sh_*, and a good agreement can be reported in Figure 5b.

While low haziness is pursued in the case of displays, such as touch panels or transparent heaters, large haze factors are desirable in solar cells since a large *HF* leads to an increase in the photon’s optical path, which results in a higher absorption within the active layer of a solar cell [62,63]. Therefore several studies have been devoted to the investigation of the haze values of MNW networks [16,64,65,66,67]. Generally speaking, it is reported that the haziness of a MNW network increases linearly with the network *amd* for a given MNW size. Haziness thus appears to drastically depend upon MNW size; for instance, Preston et al. [66] observed that AgNW with larger diameters are associated with larger haze factor values. Araki et al. demonstrated that increasing the average AgNW length allows the decrease of the haziness [65]. Therefore, the level of haziness appears as a tuneable parameter that can drastically impact the efficiency of the integration of the AgNW based transparent electrode within the final device. For instance, the AgNW used for the experiments reported in Figure 5a,b has an average diameter of 117 nm. This diameter appears to be too large for transparent heater applications. Indeed, defogging or defrosting applications (windscreens) require rather low electrical resistances and therefore a rather dense network, inducing a too large HF (≈ 10%), as shown in Figure 5b. As a result, the vision would be excessively blurred.

The previously described approach, as simple as it may seem, is in good agreement with the experimental data. However, one should also keep in mind that this is only valid approach within the percolation regime (i.e., for not too dense networks). It is also worth noting that another approach developed by Coleman uses another figure of merit (derived in the domain of thin films): the ratio between electrical and optical conductivity; this also allows for a good description of the experimental data in a broader range of network densities. In Coleman’s approach, both percolative and bulk regimes were explored [9,29,33], while here only the percolative regime has been explored. However the rather straightforward analysis presented here appears well suited to swiftly determine the network densities or post-treatments that can optimize the *FoM* of networks, as well as for assessing MNW quality and stability.

In addition to the numerous experimental approaches reported so far in the literature, a number of modelling approaches have also been explored to better understand or predict the properties of MNW networks. The seminal papers by Coleman’s group showed that for MNW networks sparse enough to display transparency over 90%, the conduction mechanisms can be understood through percolation theory [33]. Coleman et al. also analysed the data related to transmittance as a function of sheet resistance for different networks: graphene, carbon nanotubes, and metallic nanowires for which they reported a transition from bulk-like behaviour to percolation behaviour as the network density decreased [9,29]. In parallel to such modelling approaches, several recent works have been dedicated to the computation of simulated networks. For instance, O’Callaghan et al. generated simulated networks with perfectly accurate mimicking of real networks observed by scanning electron microscopy [68]. They were able to find the effective medium network that can describe the dependence of the resistance of a MNW network versus network density, electrode size, and electrode separation [68]. One of the main advantages of such methods is the possibility to disentangle the contributions from MNW junctions and the MNW themselves to the network electrical resistance. The same group also introduced a protocol which allows the calculation of the electrical performance of MNW networks [69], thanks to a combined experimental and computational approach where the geometric aspects of the network, as well as the electrical contribution of the junctions and single NWs can be taken into account all together. This can be very beneficial for a more realistic prediction of the performances of any network, with the final goal of optimizing them. In parallel, Large et al. used finite-size scaling theory to investigate AgNW networks for both experimental and simulation purposes [70]. This approach appears to be very pertinent since instead of considering the properties of MNW networks as homogeneous large area films, some applications (such as capacitive touch sensors) require device patterning. Large et al. used the finite-size scaling theory to investigate the influence of the variation of the MNW length distribution on the properties of the network [70]. This approach could have direct implications towards choosing the appropriate MNW nature and dimensions for a more efficient integration within industrial applications that require device patterning.

In parallel, while several figures of merit (FoM) have been proposed in the literature, for some applications they fail to provide a pertinent guide for fabricating an optimized network. A clear and simple example in this regard concerns solar cells: if one imagines fabricating a network composed of ultra-long MNW, we would reach both high values of transparency and electrical conductivity even for very sparse networks and this would be associated with very high values of the FoM. However, when integrated into a solar cell, the collection efficiency of the photo-generated carriers of such a sparse network would be very low in spite of its high FoM value, and thus the associated solar cell efficiency would be low. This clearly shows that an appropriate method to assess a MNW network for such an important application is still missing. Additionally, Kumar et al. [71] reported an interesting study to assess MNW based TEs for optimizing solar cell performance by considering, for instance, the influence of the active layer diffusion length of the solar cell.

## 5. Materials and Methods 

TEs discussed here were fabricated using AgNWs dispersed in isopropanol, purchased from Seashell Technology (San Diego, CA, USA) or ACS Materials (with various dimensions) and were all grown by the polyol process. In Figure 2,Figure 5 , AgNW based electrodes were fabricated by spin coating on glass substrates while in Figure 3,Figure 4 they were fabricated by spray coating. AgNW from Seashell Technology were used for the experiments reported in Figure 2,Figure 3,Figure 5, while AgNW based electrodes associated to Figure 4 were fabricated with AgNWs from ACS Materials. The density of the AgNW networks, and their associated *amd*, were calculated from the SEM images. All SEM images were obtained on an environmental FEI Quanta 250 FEG SEM (FEI, Hillsboro, OR, USA). The samples were characterized optically by a Perkin Elmer Lambda 950 UV-Visible-Near IR spectrophotometer (Perkin Elmer, Waltham, MA, USA) equipped with an integrating sphere over a wavelength range from 250 to 2500 nm. Measurements were performed on two different locations for each sample, and were then averaged.

The ITO film used in Figure 3b was commercially purchased ITO on PET from Sigma Aldrich (St. Louis, MO, USA), with an initial sheet resistance of 60 ohms per square and a film thickness of 300 nm. FTO films were deposited by spray pyrolysis in experimental conditions similar to those reported in the articles of Giusti et al. [63] and Consonni et al. [72]. The FTO film had a thickness of 250 nm and sheet resistance of 10 ohms per square.

In situ measurements of the sample’s resistance were performed with the help of a source meter Keithley 2500. The area of the AgNW networks deposited on the glass was constantly maintained with a 7 mm gap between the contact electrodes, made with silver paint. The samples were heated on a hot plate in air at a constant ramp rate of 2 °C/min from room temperature to 500 °C, while the resistance was measured in situ by using a two probe measurement setup. Sheet resistances were measured using a linear four-point probe setup (Lucas Signatone QuadPro, Pro4-440N configuration).

The Al_2_O_3_ layer was deposited using a home-made SALD system [57] with the following parameters: precursors: trimethylaluminium (TMA) and water; inert gas: nitrogen; temperature: 200 °C; distance between substrate-SALD deposition head: 150 µm; scanning speed: 10 cm/s.

## 6. Concluding Remarks and Perspectives

In addition to their extreme ease of fabrication, randomly-oriented metallic nanowire (MNW) networks are flexible efficient transparent electrodes that show great interest for several applications. They simultaneously exhibit mechanical flexibility, high optical transparency, and low electrical resistance and have been shown to be prime candidates for replacing the standard material (ITO) for a range of applications. Regarding the conduction models and the stability (thermal, electrical, chemical), MNW networks behave in a very different way compared to a thin homogeneous TCO. This is due to the specific architecture of such networks, namely with a percolating nature, and to the dimensions of the interconnected nano-objects. 

In this paper, we have briefly presented some of the important aspects of such networks for a better understanding and optimization of their physical properties. This work relates the facts that: (i) appropriate thermal annealing, as well as other post-treatments, can optimize a MNW network’s electrical resistance, (ii) a very thin coating of a transparent oxide deposited by SALD in atmospheric conditions can enhance their stability (iii) simple models can help to understand the physical properties and their interdependency and allow optimisation of the device properties. We also briefly describe how nanocomposites do not only enhance stability but also create new opportunities for the integration of MNW based transparent and/or flexible electrodes.

Finally, let us briefly discuss a few future potential directions related to MNW networks. They will very probably involve the synthesis of MNW, and the associated stability issues, using less expensive metals than silver, such as copper or metal alloys. The stability of the MNW itself still has to be addressed in spite of several demonstrations of possible improvements in the literature. The problem appears even more drastic for metals other than silver. Up-scaling MNW growth and efficient methods for fabricating MNW networks with reproducible properties at industrial scales and rates are being investigated by several companies. The results obtained will be of great importance for the future of MNW integration. 

More modelling works will probably be very useful to better understand the influence of parameters inherent to MNW networks, such as length or diameter distributions and MNW curvature (one of the rare studies of the curved MNW in network properties has been recently reported by Kumar et al. [73]). Generally speaking, it seems that each targeted application requires transparent electrodes with specific criteria of optimization. Hence, only collective thorough investigations with all concerned disciplines (chemistry, physics, material engineering, experimental and modelling approaches, etc.), from both academic and industrial partners, can subsequently lead to an efficient and large scale integration of MNW networks in commercial devices. 

## Figures and Tables

**Table 1:** A few characteristics associated to five common methods used to fabricate MNW networks. This corresponds to general indications since the main tendencies depend on experimental conditions and application requirements. Plus signs indicate better values.

Method	Homogeneity	Scalable	Cost	Examples in the Literature
Spin coating	+	+	+++	Langley [21], Lagrange [16]
Spray coating	+++	+++	++	Coskun [7]
Vacuum filtration	+++	+	+	De [28]
Drop casting	+	+	+	Gobelt [40]
Meyer rod	++	+++	++	Deng [17]
